# Nursing interventions for managing treatment-related adverse effects of targeted anti-tumor therapies in adult cancer patients: a scoping review

**DOI:** 10.3389/fphar.2026.1936674

**Published:** 2026-10-20

**Authors:** Omar Alqaisi, Suhair Al-Ghabeesh, Mohammed Dibas, Yara Alwaqfi, Ghada Yaghmour

**Affiliations:** 1 Faculty of Nursing, Al-Zaytoonah University, Amman, Jordan; 2 Department of Medicine, Faculty of Medicine and Health Sciences, An-Najah National University, Nablus, Palestine; 3 Department of Nursing, Faculty of Nursing, Salina Regional Health Center: Salina, Salina, KS, United States

**Keywords:** adverse effects, nursing interventions, oncology nursing, patient education, quality of life, scoping review, symptom management, targeted anti-tumor therapy

## Abstract

**Background:**

Targeted anti-tumor therapies, including tyrosine kinase inhibitors, antibody-drug conjugates, and immune checkpoint inhibitors, have improved clinical outcomes and survival across multiple cancers but are associated with clinically significant dermatologic, neurological, gastrointestinal, hematologic, and immune-related toxicities that compromise adherence and quality of life.

**Objectives:**

This scoping review aimed to systematically map the available evidence on nursing interventions used to prevent, monitor, and manage adverse effects associated with targeted anti-tumor therapies in adult cancer patients.

**Methods:**

Eligible studies were English-language publications conducted between 2015 and 2025 that focused on nurse-led or nursing-centered interventions addressing the adverse effects of targeted anti-tumor therapies in adult oncology populations. Data were charted using a standardized form and synthesized descriptively by intervention type and adverse-effect domain.

**Results:**

Of 742 records identified, 14 studies met the inclusion criteria. Study designs included two quasi-experimental studies and one randomized controlled trial protocol two cohort studies, seven narrative or integrative reviews, and two clinical practice guidelines. Five principal intervention categories were identified: patient education and self-management support; nurse-led oral targeted-drug management and continuity-of-care programs; home-based multidisciplinary interventions; telephone and telehealth-based toxicity monitoring; and symptom-specific supportive-care strategies. Across diverse settings, structured nursing interventions reduced toxicity burden, improved medication adherence, decreased unplanned healthcare use, and enhanced patient-reported quality of life and psychosocial outcomes.

**Conclusion:**

Nurse-led interventions may have an important and promising role in supporting the safe and effective use of targeted anti-tumor therapies in adults with cancer. Integrating structured education, continuity of care, multidisciplinary collaboration, and telehealth-enabled monitoring into routine oncology practice may optimize toxicity management and support treatment adherence. Further multicenter trials are needed to evaluate nursing protocols for antibody-drug conjugate-induced neuropathy and immune-related adverse events and to examine long-term clinical and economic outcomes.

**Systematic Review Registration:**

https://osf.io/5nvfe/identifier, 5nvfe.

## Introduction

Cancer remains a major public health challenge in the twenty-first century. According to recent GLOBOCAN data, approximately 20 million new cancer cases were diagnosed globally in 2022, with incidence projected to rise in the coming decades (Bray et al., 2024). Despite advances in treatment, the 5-year overall survival rate for patients with advanced cancer treated with conventional therapies such as chemotherapy and radiotherapy remains as low as 22%, emphasizing the urgent need for more effective and better-tolerated treatment options (Du et al., 2021). Targeted anti-tumor therapies have emerged as a transformative approach in oncology. Unlike conventional cytotoxic chemotherapy, targeted therapies selectively affect cancer cells by interfering with specific molecular pathways responsible for tumor growth, proliferation, and metastasis (Sun et al., 2025). Their primary mechanisms include inhibition of oncogenic signaling pathways, induction of apoptosis, and suppression of angiogenesis (Sun et al., 2025). The main categories of targeted agents are monoclonal antibodies, which bind with high specificity to extracellular targets, and small-molecule tyrosine kinase inhibitors (TKIs), which block intracellular kinase-dependent signaling cascades (Kay, 2006). Major drug classes in clinical use include epidermal growth factor receptor (EGFR) inhibitors (e.g., erlotinib, gefitinib, cetuximab), human epidermal growth factor receptor 2 (HER2)-directed agents (e.g., trastuzumab), vascular endothelial growth factor (VEGF) and multi-targeted TKIs (e.g., bevacizumab, sunitinib, sorafenib), mammalian target of rapamycin (mTOR) inhibitors (e.g., everolimus, temsirolimus), anaplastic lymphoma kinase (ALK) inhibitors (e.g., crizotinib, lorlatinib), and immune checkpoint inhibitors (ICIs) targeting PD-1, PD-L1, and CTLA-4 (Sun et al., 2025; Schneider et al., 2021). Across various cancer types, including non-small cell lung cancer (NSCLC), colorectal cancer, breast cancer, melanoma, and hematologic malignancies, targeted therapies have produced significant improvements in overall survival, progression-free survival, and response rates compared to conventional treatments (Du et al., 2021). For the purposes of this review, targeted anti-tumor therapies were defined as molecularly targeted agents, including TKIs, monoclonal antibodies, mTOR inhibitors, and ADCs, as well as combination regimens incorporating immune checkpoint inhibitors where relevant to toxicity management. Standalone immunotherapy studies were excluded because immune-related adverse event management has an established, independent evidence base.

Despite their therapeutic advantages, targeted anti-tumor therapies are associated with a significant spectrum of adverse effects (AEs). Although fatal adverse reactions are less common than with cytotoxic chemotherapy, the prolonged or indefinite use of targeted therapies leads to a high cumulative incidence of adverse reactions, reaching up to 80% for certain drug classes (Du et al., 2022a). A retrospective multicenter study involving 485 cancer patients receiving targeted therapy reported that 61% experienced at least one adverse drug reaction, with skin toxicities, fatigue, mucosal damage, hypertension, and gastrointestinal discomfort being the most frequent complications (Du et al., 2021). EGFR inhibitors commonly cause papulopustular (acneiform) rash in 60%–90% of patients, as well as xerosis, paronychia, and hair alterations due to inhibition of EGFR signaling in basal keratinocytes (Holcmann and Sibilia, 2015). mTOR inhibitors frequently induce aphthous-like oral ulcers, termed mTOR inhibitor-associated stomatitis (mIAS), which differ in clinical presentation and pathophysiology from conventional mucositis (Chmieliauskaite et al., 2018). Immune checkpoint inhibitors can result in immune-related adverse events (irAEs) affecting nearly any organ system, including cutaneous, gastrointestinal, hepatic, endocrine, pulmonary, neurological, and hematologic toxicities (Schneider et al., 2021). Beyond the toxicities of targeted agents, radiation-induced adverse effects such as xerostomia in head and neck cancers further compound the symptom burden, significantly impairing nutritional status, sleep quality, and overall quality of life in cancer patients (Alnaeem et al., 2025).

The adverse effects of targeted therapies have significant implications for treatment outcomes and quality of life (QoL). In a prospective study of 144 patients receiving targeted therapy in ambulatory settings, patients reported an average of four AEs over a 3-month period, with approximately 20.7% of these AEs not communicated to any healthcare professional (Roger et al., 2020). Patients experiencing adverse drug reactions were more likely to discontinue medication, alter treatment regimens, or miss doses, and exhibited higher rates of cancer recurrence and metastasis compared to those without adverse reactions (Du et al., 2021). The four psychosocial impact domains reported by Du et al. (Du et al., 2022b), limited self-management capacity, psychological distress, social participation barriers, and unmet support needs, are not adequately captured by standard clinical toxicity grading tools such as CTCAE. This underscores the importance of incorporating psychosocial screening instruments into routine nursing assessments for patients receiving EGFR-TKIs. These findings indicate that the consequences of targeted therapy-related AEs extend beyond physical discomfort, substantially impairing psychological functioning, social engagement, and treatment adherence.

Nurses play a central role in managing adverse effects associated with targeted therapies. As healthcare professionals with the most sustained patient contact throughout the cancer care continuum, nurses are well-positioned to conduct early symptom assessment, implement evidence-based interventions, deliver patient education, and provide psychosocial support (Sun et al., 2025). A systematic review of nursing interventions in cancer care demonstrated that educational, psychosocial, psychological, and coping-support interventions yield significant positive outcomes across multiple health domains, including symptom severity, fatigue, anxiety, depression, and QoL (Tuominen et al., 2019). The American Society of Clinical Oncology (ASCO) guidelines for managing immune checkpoint inhibitor-induced toxicities specify that patients and caregivers should receive timely education about immunotherapy mechanisms and the clinical profile of potential irAEs before therapy initiation and throughout treatment and survivorship (Schneider et al., 2021). Clinical nurse specialists in oncology are pivotal in structured toxicity monitoring through weekly telephone surveillance, standardized assessment protocols, and facilitation of multidisciplinary team consultations when necessary (Roger et al., 2020). However, significant challenges remain, including variability in nursing staff training, inequitable resource allocation, and a limited scope of nursing research specifically addressing targeted and immunotherapy contexts (Sun et al., 2025). The existing literature provides valuable insights into specific adverse effects and individual nursing interventions; however, critical evidence gaps remain. Studies tend to focus on single drug classes, single cancer types, or single adverse effect categories, limiting the breadth of synthesized knowledge available to clinicians (Sun et al., 2025; Du et al., 2022b). Systematic reviews have rarely focused on targeted anti-tumor therapies as a distinct treatment category with a unique adverse effect profile (Tuominen et al., 2019). Given the rapid expansion of the targeted therapy landscape, a comprehensive mapping of nursing interventions applicable to this field is urgently needed. As these therapies increasingly shift cancer treatment toward ambulatory and long-term administration, nursing management of toxicity becomes essential. Therefore, this scoping review pursued four inter-related objectives: (1) to map the types and components of nursing interventions reported for adverse effects of targeted anti-tumor therapies in adult cancer patients; (2) to characterize the adverse-effect domains addressed; (3) to describe reported clinical, psychosocial, and behavioral outcomes; and (4) to identify gaps in the evidence base that warrant future investigation.

## Materials and methods

### Study design and objectives

A scoping review was conducted to systematically map the available evidence on nursing interventions addressing adverse effects associated with targeted anti-tumor therapies in adult cancer patients. The methodology followed the framework proposed by and was reported in accordance with the Preferred Reporting Items for Systematic Reviews and Meta-Analyses extension for Scoping Reviews (PRISMA-ScR) guidelines (Arksey et al., 2005). The review protocol was prospectively registered in an international database for systematic and scoping reviews (Alqaisi, 2026). Given that nine of the 14 included sources were narrative reviews or clinical practice guidelines rather than primary studies, these were included specifically to map expert-endorsed nursing recommendations in the absence of primary interventional evidence (e.g., ADC-induced neuropathy, irAE management). Findings from primary studies and secondary/narrative sources are therefore distinguished throughout Table 1 and Results.

**TABLE 1:** Summary of studies examining nursing interventions for adverse effects associated with targeted anti-tumor therapies.

Author/Year	Purpose	Setting/Country	Sample and population	Study design	Targeted therapy context	Nursing intervention	Main findings
(Sun et al., 2025)	Map nursing role in enhancing QoL in lung cancer patients receiving targeted therapy and immunotherapy	Narrative review; China	N/A	Narrative review	Targeted therapy and immunotherapy in lung cancer	Synthesis of nursing needs: symptom management, psychosocial support, patient education	Highlights pivotal nursing role in AE management; identifies gaps in staff training, resource allocation, and nursing research scope
(Schneider et al., 2021)	Summarize ICI toxicity management and patient education based on ASCO guidelines	International guidelines	N/A	Clinical guideline/narrative review	PD-1/PD-L1 and CTLA-4 inhibitors	Pre-treatment irAE education, systematic organ-based surveillance, ASCO/SITC escalation pathways for corticosteroid initiation	Evidence-based frameworks for nurses to recognize, grade, and manage irAEs and coordinate multidisciplinary care
(Roger et al., 2020)	Investigate the frequency and characteristics of adverse events not reported to healthcare professionals among ambulatory patients receiving targeted therapies	Ambulatory oncology settings, France	144 adult cancer patients receiving targeted therapy in outpatient clinics	Prospective observational study	Various targeted therapies used in routine ambulatory practice	Clinical nurse specialist–led structured toxicity monitoring using weekly telephone contacts and standardized AE assessment forms	Patients experienced a mean of four AEs over 3 months; approximately 20.7% of AEs were not reported to any health professional, highlighting the importance of proactive nurse-led surveillance
(Du et al., 2022a)	Evaluate home-based multidisciplinary intervention on skin ADRs severity in EGFR-TKI patients	Hospital-based, home care; China	NSCLC patients on EGFR-TKIs with skin ADRs (planned n = 112)	Randomized controlled trial (protocol)	EGFR-TKIs (erlotinib, gefitinib, osimertinib, *etc.*)	12-week home-based MDT: intensive phase (health education, targeted skin-care, psychosocial and nutritional support) + maintenance phase; nurse as primary coordinator	The protocol describes planned evaluation of skin ADR severity, self-management ability, and quality of life; no trial outcome data were reported
(Diez de los Rios de la Serna et al., 2024)	Review early recognition and management of SACT-related side effects in advanced breast cancer	United Kingdom and European breast cancer services	N/A	Narrative review	Endocrine therapy, chemotherapy, targeted agents, ADCs	Nurse-led assessment, CTCAE grading, UKONS telephone triage, patient self-management support	Underlines essential role of oncology nurses in early detection, grading, and holistic toxicity management
(Dailah et al., 2024)	Provide nursing-practice overview of immunotherapy and targeted therapy in cancer	Narrative review; Saudi Arabia	N/A	Narrative review	Broad range of targeted and immune-based therapies	Nursing assessment and management of AEs, nurse-led patient education on drug mechanisms and adverse-event profiles	Emphasizes need for nurses to understand drug mechanisms and AE profiles for effective education, early detection, and care coordination
(Parreira et al., 2024)	Describe nursing role in managing TRAEs of axitinib plus IO agents in advanced RCC	International expert group	N/A	Narrative review with practice algorithms	Axitinib + avelumab/pembrolizumab (IO–TKI combination)	Nurse-oriented triage and management algorithms for diarrhea, hypertension, fatigue, rash/pruritus using CTCAE and UKONS tools	Provides nurse-friendly algorithms for early TRAE assessment, escalation, and patient education in advanced RCC
(Gao and Guo, 2025)	Evaluate structured oncology nursing interventions on chemotherapy-induced toxicities in lung cancer	Tertiary cancer hospital; China	313 lung cancer patients receiving first-line chemotherapy (intervention n = 148, control n = 165)	Retrospective cohort	Chemotherapy ± targeted agents	Structured oncology nursing: toxicity education, scheduled telephone follow-up, early triage, individualized supportive care plans	Reduced grade ≥2 neutropenia and CINV, fewer unplanned emergency visits and treatment delays, lower anxiety, higher satisfaction
(Zhang and Lian, 2025)	Evaluate oral targeted drug management program led by specialist nurses in NSCLC out-of-hospital care	First Affiliated Hospital of Suzhou University; China	80 NSCLC patients on icotinib (intervention n = 40, control n = 40)	Quasi-experimental (RCT method, random number table)	EGFR-TKI (icotinib)	Nurse-led program: patient filing, medication record cards, ADR registration forms, weekly SMS health guidance, re-examination reminders	Reduced missed medication events, improved adherence, lower ADR incidence, better QoL at 6 months
(Min et al., 2023)	Examine impact of continuous care led by specialist nurses on QoL after targeted therapy in NSCLC	Three hospitals; China	NSCLC patients on targeted therapy post-discharge	Quasi-experimental continuous-care study	EGFR-targeted therapy	Nurse-led continuous care: education, lifestyle/exercise counselling, sleep-hygiene guidance, ongoing follow-up contacts	Improved QLQ-C30 and SF-36 scores, reduced anxiety/depression, decreased adverse-reaction incidence vs. routine care
(Zhang et al., 2024)	Summarize nursing management of peripheral neuropathy induced by antibody–drug conjugates	Oncology nursing/breast cancer center; China	N/A	Clinical practice review	Antibody–drug conjugates (ADCs)	Risk-factor assessment, validated neuropathy scales, functional/gait screening, patient education, PT/OT referral, fall-prevention strategies	Defines key nursing responsibilities in early identification, functional management, and safety monitoring for ADC-induced PN
(Barber, 2019)	Summarize nursing management of immune checkpoint inhibitor toxicities	International review	N/A	Narrative review	PD-1/PD-L1 and CTLA-4 inhibitors	Patient education on irAE warning signs, systematic monitoring, escalation protocols	Provides nursing framework for irAE recognition and multidisciplinary escalation
(Shankar et al., 2022)	Review supportive care and nursing considerations for ICI-related adverse events	International review	N/A	Narrative review	Immune checkpoint inhibitors	Structured nursing monitoring, patient education, early triage and reporting of irAEs	Highlights nursing role in sustaining treatment by early irAE detection and coordinated management
(Califano et al., 2015)	Provide expert consensus on management of AEs from EGFR-TKI therapy in clinical practice	United Kingdom oncology centers	N/A	Expert consensus/clinical practice review	EGFR-TKIs	Nurse-led skin-care protocols, prophylactic moisturizers, early CTCAE grading, patient education on self-care and trigger avoidance	Defines standardized nursing strategies for prevention and management of EGFR-TKI dermatologic toxicities

EGFR-TKI: epidermal growth factor receptor tyrosine kinase inhibitor; NSCLC: Non-Small Cell Lung Cancer; QoL: quality of life; QLQ-C30: Quality of Life Questionnaire–Core 30; SF-36: Short Form-36, Health Survey; CINV: Chemotherapy-Induced Nausea and Vomiting; ADC: Antibody-Drug Conjugate; PD-1: Programmed Death Receptor-1; PD-L1: Programmed Death Ligand-1; CTCAE: common terminology criteria for adverse events; UKONS: united kingdom oncology nursing society; IO: Immuno-Oncology; NA: not applicable.

The review was guided by the following research question: *What is the nature, scope, and reported impact of nursing interventions used to prevent, monitor, or manage adverse effects associated with targeted anti-tumor therapies in adult cancer* patients?

### Search strategy

A comprehensive literature search was performed across four electronic databases: PubMed/MEDLINE, Scopus, ScienceDirect, and CINAHL. Following peer-review feedback, an additional search was conducted in Web of Science using the same search strategy (Supplementary File S1). This yielded 85 records, of which 22 remained after duplicate removal against the original four databases. Full-text review of six potentially relevant records identified none meeting all eligibility criteria, primarily due to protocol-only status without reported outcomes or non-exclusive focus on molecularly targeted therapy populations. Searches were conducted between January and February 2026. The PubMed search strategy combined MeSH headings and free-text keywords: (“neoplasms”[MeSH] OR “cancer”) AND (“targeted therapy” OR “tyrosine kinase inhibitor” OR “monoclonal antibody” OR “antibody-drug conjugate”) AND (“nursing intervention” OR “oncology nursing” OR “nurse-led”) AND (“adverse effects” OR “toxicity” OR “symptom management”). Equivalent terms and subject headings were adapted for Scopus, ScienceDirect, and CINAHL. No date restrictions were applied; publications from 2015 to 2025 were included based on clinical relevance to contemporary targeted agents. MeSH terms were used in PubMed; controlled vocabulary was applied where available in other databases.

The complete search strategy for each database is provided in Supplementary File S1. Additionally, reference lists of included studies and relevant review articles were hand-searched to identify further eligible records.

### Eligibility criteria

Studies were included if they met the following criteria: (1) focused specifically on nursing interventions, nurse-led care, nursing management, or structured supportive nursing strategies; (2) involved adult cancer patients receiving molecularly targeted anti-tumor therapies (monoclonal antibodies, tyrosine kinase inhibitors, mTOR inhibitors, or BRAF-targeted agents), administered alone or in combination with immune checkpoint inhibitors; (3) addressed adverse effects, symptom burden, toxicity prevention, toxicity monitoring, or adverse event management related to targeted therapy; (4) reported clinical, psychosocial, behavioral, or patient-reported outcomes for primary studies, or evidence-based nursing recommendations and management algorithms for narrative reviews and clinical practice guidelines; (5) were published in English.

Studies were excluded if they: (1) were not focused on targeted anti-tumor therapy-related adverse effects; (2) did not include a distinct nursing component; (3) focused exclusively on immune checkpoint inhibitors or CAR-T therapy, without any targeted-therapy component (e.g., TKIs, mTOR inhibitors, or ADCs), as standalone immunotherapy studies were considered outside the scope of this review, given their independent evidence base. Note: combination regimens pairing an ICI with a molecularly targeted agent (e.g., axitinib plus pembrolizumab) remained eligible; only ICI-monotherapy studies were excluded; (4) focused exclusively on physician-led or pharmacological management without a nursing intervention component; (5) included pediatric populations only; or (6) were conference abstracts, editorials, letters, or publications lacking sufficient methodological detail for data extraction.

As a scoping review, the methodological objective was to map the breadth and nature of the available evidence across diverse study designs, rather than to evaluate intervention effectiveness through pooled quantitative synthesis. Narrative reviews and clinical practice guidelines were deliberately included alongside primary studies because dedicated interventional trials were unavailable for several clinically important domains, particularly ADC-induced peripheral neuropathy and immune-related adverse events. Table 1 explicitly identifies the design of each source, and findings from primary studies are distinguished from those of narrative/guideline sources throughout the Results and Discussion sections.

### Study selection and data extraction

Two authors (O.A. and S.A.) independently screened the titles and abstracts of all retrieved records against the predefined eligibility criteria, followed by full-text review of potentially relevant studies. Any discrepancies were resolved through discussion with the third author (M.D.). The study selection process was documented using a PRISMA-ScR flow diagram (Figure 1). A completed PRISMA-ScR reporting checklist will be provided in the Supplementary Materials (Supplementary Table S2).

**FIGURE 1:**
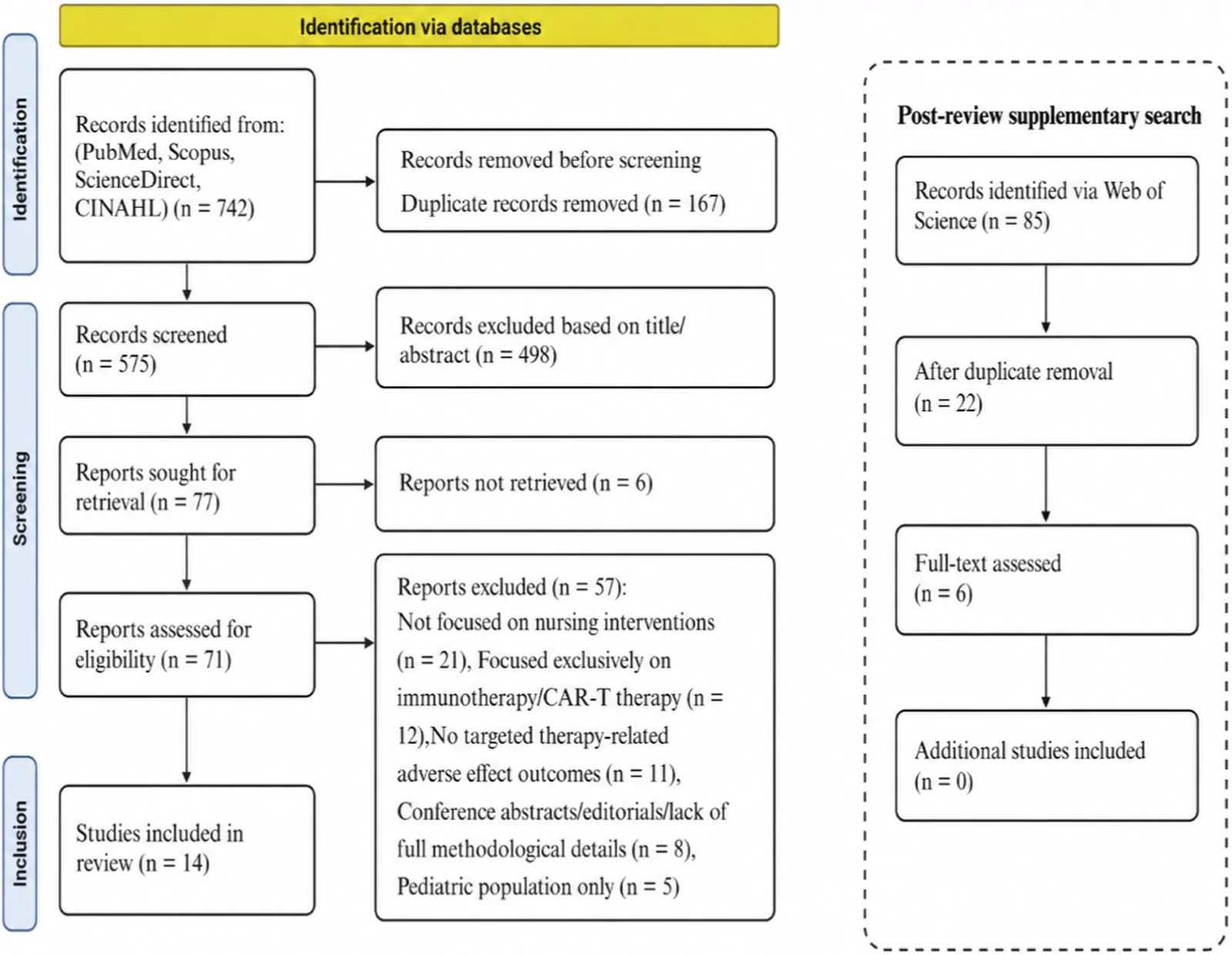
Preferred reporting items for systematic review and meta-analysis extension for Scoping Reviews (PRISMA-ScR).

Data were extracted using a standardized data-charting form developed specifically for this review. Extracted items included author/reference, study purpose, setting, sample size, study design, targeted therapy context, type of nursing intervention, adverse effects addressed, and main findings. In accordance with scoping review reporting practices, the detailed characteristics of the included studies will be presented in Table 1. Data charting was performed independently by two reviewers (O.A. and S.A.) and any discrepancies were resolved through discussion with the third reviewer (F.H.).

### Critical appraisal and data synthesis

In accordance with PRISMA-ScR guidance for scoping reviews, a formal methodological quality appraisal or risk-of-bias assessment of the included studies was not conducted. The aim was to map the range, characteristics, and focus of the available evidence rather than to exclude studies based on methodological hierarchy.

Extracted data were synthesized descriptively and organized by nursing intervention type and the adverse effects addressed. Where appropriate, findings were grouped into broad thematic categories, including skin toxicity management, patient education and self-management support, telephone or digital follow-up, psychosocial support, and multidisciplinary or structured nursing-led monitoring models. This approach provided a structured overview of the current evidence base and facilitated the identification of gaps requiring future research.

## Results

### Study selection

A total of 742 records were identified through searches of PubMed/MEDLINE, Scopus, ScienceDirect, and CINAHL. An updated Web of Science search conducted post-review identified 85 additional records; after duplicate removal and full-text screening, no additional studies met the eligibility criteria (see Search Strategy). Following the removal of 167 duplicates, 575 unique records underwent title and abstract screening. Of these, 498 were excluded for not meeting eligibility criteria, such as lacking a focus on targeted therapy, absence of a nursing component, or irrelevance to adverse-effect management. Seventy-seven full-text reports were sought for retrieval; however, six could not be obtained in full text despite repeated attempts. These six records were therefore excluded from the review.

Seventy-one full-text articles were assessed for eligibility. Of these, 57 were excluded for the following reasons: lack of focus on nursing interventions (n = 21), exclusive focus on immunotherapy or CAR-T therapy without a targeted-therapy component (n = 12), absence of targeted-therapy-related adverse-effect outcomes (n = 11), being conference abstracts, editorials, or lacking sufficient methodological detail (n = 8), and inclusion of only pediatric populations (n = 5). Fourteen studies fulfilled all inclusion criteria and were incorporated into the final synthesis. The study selection process is depicted in the PRISMA-ScR flow diagram (Figure 1).

### Characteristics of included studies

The fourteen included studies were published between 2015 and 2025 and represented a range of geographical regions, including China, Italy, other European countries, North America, and the Middle East (Sun et al., 2025; Du et al., 2022a; Diez de los Rios de la Serna et al., 2024; Dailah et al., 2024; Parreira et al., 2024). Study designs encompassed randomized controlled trials, quasi-experimental before-and-after studies, prospective and retrospective cohort studies, as well as narrative or integrative reviews with a clearly defined nursing-intervention component (Du et al., 2022a; Diez de los Rios de la Serna et al., 2024). Sample sizes varied from small pilot interventions with fewer than 40 participants to multicenter randomized trials and large retrospective cohorts with more than 300 patients (Gao and Guo, 2025; Zhang and Lian, 2025).

The targeted anti-tumor agents examined across the studies included epidermal growth factor receptor tyrosine kinase inhibitors (EGFR-TKIs; such as erlotinib, gefitinib, afatinib, osimertinib, icotinib), multi-targeted TKIs (such as axitinib, sunitinib, sorafenib, lenvatinib, cabozantinib), antibody-drug conjugates, and immune checkpoint inhibitors targeting PD-1 or PD-L1 (Sun et al., 2025; Dailah et al., 2024). The cancer types addressed included non-small cell lung cancer (NSCLC), breast cancer, renal cell carcinoma, colorectal cancer, melanoma, and other solid tumors (Du et al., 2022a; Parreira et al., 2024). Most interventions were implemented in outpatient oncology clinics or home-based settings, while some were hospital-based or utilized mixed models within comprehensive cancer centers (Du et al., 2022a; Gao and Guo, 2025). Table 1 provides a structured overview of the included studies.

With respect to intervention categories, patient education and self-management support were addressed in 11 of the 14 included studies; telehealth and telephone-based monitoring in 5; multidisciplinary home-based interventions in 3; and nurse-led continuity-of-care programs in 4. Regarding toxicity domains, dermatologic toxicity was the focus of nine studies, gastrointestinal toxicity of 6, peripheral neuropathy of 2, immune-related adverse events of 4, and systemic/hematologic toxicity of three studies.

### Categories of nursing interventions

Analysis of 14 studies revealed that nursing interventions for managing adverse effects of targeted anti-tumor therapies were primarily classified as education-focused interventions, nurse-led oral targeted-drug management and continuity-of-care programs, multidisciplinary nurse-coordinated interventions, telephone- and telehealth-based toxicity monitoring, and symptom-specific supportive-care strategies (Sun et al., 2025; Du et al., 2022a; Dailah et al., 2024).

Education-focused interventions involved structured instruction regarding treatment mechanisms, anticipated toxicities, early warning signs, and self-care behaviors. These interventions were typically delivered by oncology nurses through face-to-face sessions, printed materials, and electronic resources, and were sometimes reinforced *via* online platforms such as WeChat (Sun et al., 2025; Diez de los Rios de la Serna et al., 2024). Nurse-led oral targeted-drug management and continuity-of-care programs for patients receiving EGFR-TKIs, particularly icotinib, incorporated individualized medication plans, execution record cards, adverse-reaction diaries, and regular follow-up *via* telephone or social media to improve adherence and toxicity management (Zhang and Lian, 2025; Min et al., 2023).

Home-based multidisciplinary interventions coordinated by nurses, particularly for EGFR-TKI-related skin adverse drug reactions, integrated health education, intensive home and telephone follow-up, standardized skin-care protocols, and psychosocial and nutritional support within a structured 12-week protocol (Du et al., 2022a). Additionally, several studies assessed telephone- and telehealth-based toxicity monitoring, where oncology nurses conducted scheduled phone calls or remote assessments using patient-reported outcomes to detect emerging adverse events early and guide timely adjustments in supportive care (Gao and Guo, 2025). Narrative and practice-oriented reviews further described symptom-specific nursing strategies for dermatologic toxicities, peripheral neuropathy, and fatigue in patients receiving targeted therapies and IO–TKI combinations, highlighting the use of standardized triage tools and evidence-based management algorithms (Parreira et al., 2024; Zhang et al., 2024; Barber, 2019; Shankar et al., 2022). The relationships between the identified adverse effect domains, nursing intervention categories, and patient outcomes are illustrated in Figure 2. Building on this categorization, we propose an integrated three-stage nursing care model spanning the treatment continuum: (1) pre-treatment risk stratification, including baseline toxicity-risk assessment and anticipatory patient education; (2) active monitoring and self-management support, encompassing telehealth surveillance, symptom diaries, and structured self-care protocols; and (3) escalation and multidisciplinary coordination, involving CTCAE-based triage, specialist referral, and coordinated dose-modification decisions. This staged model reframes the identified interventions as sequential, interdependent components of care rather than isolated categories.

**FIGURE 2:**
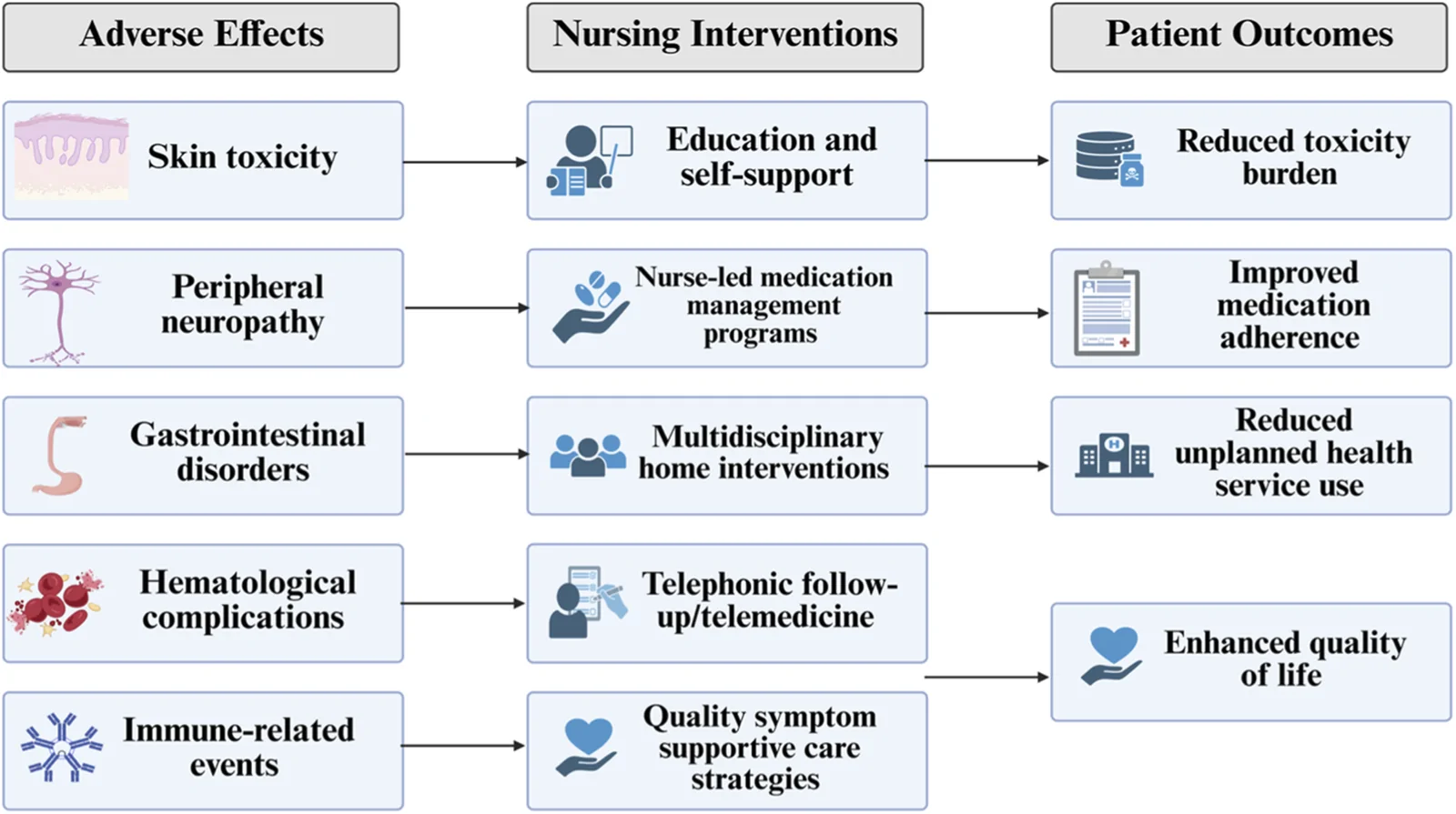
Conceptual framework illustrating the relationships between adverse effects of targeted anti-tumor therapies, nurse-led intervention categories, and patient outcomes synthesized from the included studies. Arrows denote thematic associations reported across the included literature rather than empirically established causal pathways, given the descriptive nature of this scoping review. ADC: Antibody-Drug Conjugate; EGFR-TKI: Epidermal Growth Factor Receptor Tyrosine Kinase Inhibitor; ICI: Immune Checkpoint Inhibitor; QoL: Quality of Life.

### Adverse effects targeted by nursing interventions

The nursing interventions identified in this scoping review addressed a wide range of adverse effects associated with targeted anti-tumor therapies. The most frequently targeted domains included dermatologic toxicity, peripheral neuropathy, gastrointestinal toxicity, systemic and hematologic toxicity, and immune-related adverse events (Parreira et al., 2024; Gao and Guo, 2025; Zhang et al., 2024; Califano et al., 2015).

Table 2 demonstrates that most nursing interventions address dermatologic and gastrointestinal toxicities, which are highly prevalent and significantly affect adherence among patients receiving EGFR-TKIs, multi-targeted TKIs, and IO–TKI combinations (Sun et al., 2025; Parreira et al., 2024; Califano et al., 2015). Although peripheral neuropathy and immune-related adverse events are less frequently the primary focus of interventional trials, they are thoroughly discussed in clinical practice reviews and guidelines. This suggests substantial opportunities to develop more structured nursing protocols in these areas (Schneider et al., 2021; Zhang et al., 2024).

**TABLE 2:** Adverse effects targeted by nursing interventions in the included studies.

Adverse effect domain	Typical toxicities	Targeted therapy classes	Key nursing intervention components	Representative references
Dermatologic toxicities	Acneiform rash, xerosis, pruritus, paronychia, hand–foot syndrome, photosensitivity	EGFR-TKIs, multi-targeted TKIs, antibody–drug conjugates, IO–TKI combinations	Prophylactic moisturizers and emollients, sun-protection counselling, avoidance of friction and extreme temperatures, early CTCAE-based grading, standardized skin-care protocols, timely referral to dermatology, patient education on self-care	(Sun et al., 2025; Du et al., 2022a; Diez de los Rios de la Serna et al., 2024; Califano et al., 2015)
Peripheral neuropathy	Distal numbness, tingling, burning pain, weakness, gait disturbance, falls	Antibody–drug conjugates, some TKIs	Baseline risk assessment, use of neuropathy scales (e.g., Total Neuropathy Score), functional screening of gait and fine motor skills, safety and fall-prevention counselling, referral to physical and occupational therapy, coordination with physicians on dose modifications	(Zhang et al., 2024)
Gastrointestinal toxicity	Diarrhea, nausea, vomiting, stomatitis, mTOR-inhibitor-associated stomatitis	EGFR-TKIs, VEGF-TKIs, mTOR inhibitors, IO–TKI regimens	Dietary counselling and hydration advice, education on appropriate use of anti-emetics and anti-diarrheals, mouth-care and oral-hygiene protocols, structured triage using CTCAE and UKONS tools, telephone follow-up for red-flag symptoms	(Diez de los Rios de la Serna et al., 2024; Parreira et al., 2024; Gao and Guo, 2025)
Systemic and hematologic toxicity	Fatigue, anemia, neutropenia, infection risk	Chemotherapy plus targeted agents, IO–TKI combinations	Patient education on infection-prevention and energy-conservation strategies, routine laboratory monitoring, early triage for fever or severe fatigue, coordination of supportive medications (e.g., G-CSF), symptom-specific management algorithms	(Gao and Guo, 2025)
Immune-related adverse events	Endocrinopathies (hypothyroidism, hypophysitis), colitis, hepatitis, pneumonitis, cutaneous irAEs	PD-1/PD-L1 inhibitors, CTLA-4 inhibitors	Education on immunotherapy mechanisms and irAE warning signs, systematic review-of-systems assessments, close monitoring for early irAE symptoms, adherence to ASCO and SITC escalation pathways for corticosteroid initiation and treatment interruption, coordination of multidisciplinary care	(Sun et al., 2025; Schneider et al., 2021; Barber, 2019; Shankar et al., 2022)

EGFR-TKI: epidermal growth factor receptor tyrosine kinase inhibitor; TKI: tyrosine kinase inhibitor; ADC: Antibody-Drug Conjugate; IO: Immuno-Oncology; VEGF: vascular endothelial growth factor; mTOR: mammalian target of rapamycin; CTCAE: common terminology criteria for adverse events; UKONS: united kingdom oncology nursing society; PD-1: Programmed Death Receptor-1; PD-L1: Programmed Death Ligand-1; CTLA-4: Cytotoxic T-Lymphocyte-Associated Protein 4; ASCO: american society of clinical oncology; SITC: society for immunotherapy of cancer; G-CSF: Granulocyte Colony-Stimulating Factor; irAE: Immune-Related Adverse Event.

### Outcomes associated with nursing interventions

Although study designs and outcome measures varied, the reviewed literature consistently demonstrated that structured nursing interventions reduced toxicity burden, improved treatment adherence, optimized healthcare utilization, and enhanced patient-reported outcomes (Du et al., 2022a; Gao and Guo, 2025; Zhang and Lian, 2025). Nurse-led oral targeted drug management and continuous care programs for patients with NSCLC receiving EGFR-TKIs significantly reduced missed doses, lowered the incidence of moderate to severe skin reactions and diarrhea, and improved medication compliance scores compared to conventional nursing approaches (Zhang and Lian, 2025; Min et al., 2023). Du et al. (2022a) described a protocol for a randomized controlled trial evaluating a home-based multidisciplinary intervention for EGFR-TKI-related skin adverse reactions. As no outcome data were reported, the effectiveness of the intervention remains to be established.

Structured oncology nursing interventions targeting chemotherapy-related toxicities in lung cancer were linked to lower rates of grade 2 or higher neutropenia and chemotherapy-induced nausea and vomiting, as well as fewer unplanned emergency visits and treatment delays. These interventions also resulted in reduced anxiety and increased patient satisfaction (Gao and Guo, 2025). Continuous care models and nurse-led supportive interventions for patients undergoing targeted therapy were associated with improvements in global quality-of-life indices and functional domains, including physical, social, and role functioning. These interventions also led to reductions in anxiety and depression scores as measured by validated symptom scales (Min et al., 2023). Additionally, narrative reviews and clinical guidelines emphasized that proactive, structured nursing involvement in toxicity education, monitoring, triage, and psychosocial care is critical for maintaining dose intensity and optimizing outcomes in adults receiving targeted therapies and immunotherapies (Sun et al., 2025; Dailah et al., 2024; Barber, 2019; Shankar et al., 2022).

## Discussion

This scoping review suggests that nursing interventions for patients receiving targeted anti-tumor therapies are multidimensional, spanning patient education, structured drug management and continuity-of-care programs, home-based multidisciplinary models, telehealth-enabled toxicity monitoring, and symptom-specific supportive care strategies. These findings highlight the central coordinating role of oncology nurses across the treatment continuum in targeted-therapy settings.

### Patient education and self-management

Patient education emerged as the most consistently reported nursing intervention across the included studies. Structured educational programs delivered by oncology nurses through face-to-face sessions, printed materials, and digital platforms such as WeChat were associated with improved patient knowledge of adverse effects, enhanced self-management capabilities, and reduced rates of treatment non-adherence. The improvements in adherence observed in nurse-led oral targeted-drug management programs are consistent with broader evidence on self-management support in chronic disease, where structured nurse education and follow-up can substantially reduce unintentional non-adherence and improve symptom control (Alqaisi et al., 2026). These findings are consistent with the broader literature, which demonstrates that patients who receive structured pre-treatment counseling and ongoing educational support are better equipped to recognize and manage adverse reactions at home, thereby reducing the risk of dose reduction or premature treatment discontinuation.

Caregiver involvement and teach-back methods were highlighted as effective strategies for reinforcing comprehension across diverse health literacy levels (Sun et al., 2025; Diez de los Rios de la Serna et al., 2024). The integration of mobile health platforms and electronic adverse-event monitoring tools further extended the reach of nurse-led educational interventions in outpatient settings, enabling real-time communication between patients and clinical teams. These approaches are particularly relevant given that approximately 20.7% of adverse events experienced by patients receiving targeted therapies in ambulatory settings are not communicated to any healthcare professional (Roger et al., 2020). The certainty of this evidence is limited, as most supporting studies are narrative reviews without primary outcome data (Sun et al., 2025; Dailah et al., 2024), while only Du et al. and Zhang and Lian provide experimental designs with control groups (Du et al., 2022a; Zhang and Lian, 2025). Clinical applicability across different targeted-therapy classes therefore remains uncertain.

### Nurse-led continuity-of-care programs

Continuous care programs guided by specialist nurses for patients with NSCLC receiving EGFR-targeted therapy demonstrated significant improvements in quality-of-life scores across multiple functional domains, meaningful reductions in anxiety and depression scores, and decreased adverse-reaction incidence compared with routine nursing care (Min et al., 2023). The benefits observed with structured nurse-led continuity-of-care programs, including fewer unplanned emergency visits and reduced treatment delays, align with international trials of remote symptom monitoring, where proactive nurse follow-up has been shown to decrease hospitalization and improve survival in oncology patients. These findings support integrating telephone- and telehealth-based monitoring as a standard component of targeted-therapy pathways. These findings underscore the clinical value of extending professional nursing oversight beyond institutional boundaries, particularly for patients on oral targeted agents in outpatient and home-based settings where intermittent in-person monitoring is insufficient. This finding derives from a single quasi-experimental study in one national context (Min et al., 2023), limiting consistency assessment and generalizability to other healthcare systems.

### Multidisciplinary home-based and telephone-based interventions

The home-based multidisciplinary intervention protocol for EGFR-TKI-related skin adverse drug reactions described by (Du et al., 2022a). integrated oncologists, dermatologists, pharmacists, nutritionists, and specialist nurses within a 12-week structured framework. The multidisciplinary approach was justified by the recognition that dermatologic toxicities, including papulopustular rash, xerosis, paronychia, and hand-foot syndrome, involve cutaneous, nutritional, psychological, and pharmacological dimensions that exceed the scope of single-discipline care. Given that approximately 80% of patients receiving EGFR-TKIs develop skin adverse reactions within the first 3 months of treatment and that nearly one-third discontinue therapy because of these toxicities, the absence of standardized, nurse-led skin-care protocols in many settings represents a critical systems-level gap. Multidisciplinary home-based and telephone-based interventions may help close this gap by ensuring early identification and timely management of dermatologic toxicities. As this evidence rests on a single RCT protocol rather than completed trial results (Du et al., 2022a), conclusions about clinical effectiveness remain provisional pending the publication of outcome data.

### Peripheral neuropathy, immune-related adverse events, and psychosocial outcomes

Beyond dermatologic and gastrointestinal toxicities, this review identified emerging nursing frameworks for managing ADC-induced peripheral neuropathy and immune checkpoint inhibitor-related adverse events (Zhang et al., 2024) detailed nursing responsibilities for baseline neurological assessment, validated application of a neuropathy scale, functional screening, fall-prevention education, and rehabilitation referral for patients receiving ADCs. For immune-related adverse events, clinical guidelines and reviews consistently emphasized the nurse’s role in pre-treatment irAE education, systematic organ-based symptom surveillance, and adherence to ASCO and SITC escalation pathways for corticosteroid initiation and treatment interruption (Schneider et al., 2021; Barber, 2019; Shankar et al., 2022).

Across all intervention categories and study designs, structured nursing programs consistently improved patient psychosocial outcomes alongside their clinical effects, yielding reductions in anxiety and depression scores and higher patient satisfaction, a finding that reflects the integrated physical and psychological burden of targeted therapy toxicities (Du et al., 2022a; Min et al., 2023). A qualitative study involving patients with NSCLC receiving EGFR-TKI-related skin adverse reactions identified four principal impact domains: limited self-management ability, psychological and emotional challenges, barriers to social participation, and unmet needs for social support (Du et al., 2022b). These findings confirm that psychosocial support is not ancillary but constitutes a core dimension of effective nursing intervention in targeted therapy populations. This body of evidence is derived entirely from narrative reviews and clinical guidelines rather than interventional trials, meaning the effectiveness of these nursing frameworks for ADC-induced neuropathy and irAE management remains theoretically grounded but empirically untested.

### Evidence gaps

Despite the convergence of positive findings, this review identified several significant evidence gaps. Most included studies focused on a single drug class (predominantly EGFR-TKIs), a single cancer type (predominantly NSCLC), or a single adverse-effect category (predominantly dermatologic toxicity), with limited evidence addressing combination IO–TKI regimens or the broader polysymptom burden characteristic of real-world clinical practice (Sun et al., 2025; Du et al., 2022b). Dedicated interventional trials examining nursing management of ADC-induced peripheral neuropathy and immunotherapy-related irAEs were conspicuously absent. The overrepresentation of studies from Chinese tertiary cancer centers limits the generalizability of findings to other healthcare systems with differing resource profiles, staffing models, and cultural contexts (Sun et al., 2025). Furthermore, cost-effectiveness data and long-term clinical outcomes, including progression-free survival, overall survival, and sustained treatment adherence, were rarely reported, representing critical gaps for future research.

### Implications for practice

The findings of this scoping review have significant implications for oncology nursing practice, education, and research. In clinical settings, the evidence supports the systematic incorporation of structured nurse-led interventions, including pre-treatment toxicity education, scheduled telephone or digital follow-up, individualized skin-care and gastrointestinal management protocols, and comprehensive psychosocial assessment into standard care pathways for patients undergoing targeted anti-tumor therapies. Oncology nurses should utilize standardized toxicity assessment tools consistent with CTCAE version 5.0, individualized medication management resources, and explicit escalation protocols to enable early identification of toxicity and prompt multidisciplinary consultation. The findings also underscore the importance of nurse-led multidisciplinary coordination, especially in addressing dermatologic and neuropathic adverse effects that involve cutaneous, nutritional, and psychological domains and extend beyond the capacity of single-discipline care.

From an educational perspective, the identified deficiencies in nursing staff training and the limited breadth of nursing research in targeted and immunotherapy contexts (Sun et al., 2025) indicate a need for the development of structured oncology nursing competency frameworks. These frameworks should include up-to-date knowledge of targeted therapy mechanisms and adverse-effect profiles, proficiency in validated assessment instruments such as CTCAE and neuropathy scales, and familiarity with international management guidelines from ASCO, SITC, and ESMO. At the health system and policy levels, investment in appropriate nurse-to-patient staffing ratios in outpatient oncology settings and the establishment of standardized national nursing protocols for managing targeted-therapy adverse effects are justified, given the demonstrated clinical and healthcare utilization benefits of structured nursing programs.

### Implementation challenges and organizational implications

Translating these findings into practice faces several barriers. Nurse-to-patient staffing ratios in outpatient oncology settings, documented as inadequate in several included sources, constrain the feasibility of intensive telephone- and home-based monitoring models. Variability in oncology nursing training regarding targeted-therapy pharmacology further limits standardized implementation. The concentration of evidence in Chinese tertiary cancer centers raises concerns about transferability to healthcare systems with different staffing models, reimbursement structures, and scope-of-practice regulations. Future research priorities should include cost-effectiveness evaluations, multicenter trials beyond single-nation contexts, and implementation science frameworks to identify facilitators and barriers to the adoption of nurse-led toxicity-monitoring protocols.

### Limitations

Several limitations of this scoping review should be acknowledged when interpreting its findings. Due to the nature of the scoping review methodology, no formal risk-of-bias assessment or quality appraisal was performed for the included studies; as a result, the evidence base comprises studies with varying methodological rigor, including randomized controlled trials and quasi-experimental studies, as well as cohort studies, narrative reviews, and expert consensus documents. The search strategy was limited to four electronic databases and English-language publications, which may have excluded relevant evidence, particularly from non-English-language sources in other high-burden regions. The heterogeneity of included studies in terms of design, intervention components, targeted therapy classes, cancer types, and outcome measures prevented formal quantitative synthesis and reduces the precision of cross-study comparisons. The predominant emphasis on EGFR-TKIs and NSCLC restricts generalizability to the broader population of patients receiving targeted anti-tumor therapies, and the short follow-up periods of three to 6 months in most intervention studies are inadequate for assessing long-term clinical outcomes. Additionally, potential publication bias cannot be ruled out, as studies with positive findings are more likely to be published, and the lack of a grey literature search may have resulted in the omission of relevant unpublished program evaluations and clinical practice guidelines. Additionally, the inclusion of narrative reviews and guidelines may have introduced overlap with primary evidence.

## Conclusion

The present review offers a comprehensive overview of nursing interventions implemented to prevent, monitor, and manage adverse effects associated with targeted anti-tumor therapies in adult cancer patients. Analysis of 14 studies published between 2015 and 2025 indicates that structured nurse-led programs, including patient education and self-management support, continuity-of-care models, home-based multidisciplinary interventions, and telehealth-based toxicity monitoring, consistently produced clinical and psychosocial benefits across various targeted-therapy settings. These benefits encompass reduced toxicity severity, improved medication adherence, fewer unplanned healthcare encounters, and enhanced quality of life, underscoring the essential role of oncology nurses in addressing the complex adverse-effect burden of contemporary targeted therapies. However, the current evidence base exhibits several notable limitations. Most studies were conducted in single institutions or primarily within Chinese tertiary cancer centers, which restricts the generalizability of findings across diverse healthcare systems. Evidence gaps remain for several targeted therapy classes, particularly mTOR inhibitors and combination IO–TKI regimens, and the evidence base for ADC-specific nursing protocols is limited to a single clinical practice review, with no interventional trial data. Furthermore, there is a lack of long-term follow-up data. Future research should focus on developing and evaluating structured nursing protocols through large-scale, multicenter randomized controlled trials tailored to targeted therapy populations. Special emphasis is needed on intervention models addressing ADC-induced peripheral neuropathy and immunotherapy-related adverse events, where Level I evidence remains lacking. Incorporating validated digital health technologies, such as mobile applications, electronic patient-reported outcome platforms, and telehealth monitoring systems, into nurse-led care models represents a promising and underexplored area. Furthermore, establishing standardized oncology nursing competency frameworks that integrate knowledge of targeted therapy mechanisms and proficiency with internationally validated toxicity assessment tools is urgently needed. Such frameworks would help reduce variability in the quality of nursing care and optimize patient outcomes across global oncology settings.

## Data Availability

The original contributions presented in the study are included in the article/Supplementary Material, further inquiries can be directed to the corresponding author.

## Glossary

ADC: Antibody-Drug Conjugate
AE: Adverse Effect
ADR: Adverse Drug Reaction
ALK: Anaplastic Lymphoma Kinase
ASCO: American Society of Clinical Oncology
BRAF: B-Raf Proto-Oncogene, Serine/Threonine Kinase
CAR-T: Chimeric Antigen Receptor T-cell
CINAHL: Cumulative Index to Nursing and Allied Health Literature
CINV: Chemotherapy-Induced Nausea and Vomiting
CIPN: Chemotherapy-Induced Peripheral Neuropathy
CTCAE: Common Terminology Criteria for Adverse Events
CTLA-4: Cytotoxic T-Lymphocyte-Associated Protein 4
EGFR: Epidermal Growth Factor Receptor
EGFR-TKI: Epidermal Growth Factor Receptor Tyrosine Kinase Inhibitor
ESMO: European Society for Medical Oncology
G-CSF: Granulocyte Colony-Stimulating Factor
HER2: Human Epidermal Growth Factor Receptor 2
ICI: Immune Checkpoint Inhibitor
IO: Immuno-Oncology
irAE: Immune-Related Adverse Event
MDT: Multidisciplinary Team
mIAS: mTOR Inhibitor-Associated Stomatitis
mTOR: Mammalian Target of Rapamycin
NCI: National Cancer Institute
NSCLC: Non-Small Cell Lung Cancer
PD-1: Programmed Cell Death Protein 1
PD-L1: Programmed Death Ligand-1
PN: Peripheral Neuropathy
PRISMA-ScR: Preferred Reporting Items for Systematic Reviews and Meta-Analyses Extension for Scoping Reviews
PRO: Patient-Reported Outcome
QoL: Quality of Life
QLQ-C30: Quality of Life Questionnaire–Core 30 (EORTC)
RCC: Renal Cell Carcinoma
SF-36: Short Form-36 Health Survey
SITC: Society for Immunotherapy of Cancer
TKI: Tyrosine Kinase Inhibitor
TNS: Total Neuropathy Score
UKONS: United Kingdom Oncology Nursing Society

## References

[B1] Alnaeem M. M. Darwish S. K. Nashwan A. J. (2025). Radiation-induced xerostomia in head and neck cancers: a comprehensive review for burden and impact on nutrition, sleep, and quality of life. Discov. Med. 2 (1), 143. 10.1007/s44337-025-00362-1

[B2] Alqaisi O. M. (2026). Nursing interventions for adverse effects associated with targeted anti-tumor therapies in adult cancer patients: a scoping review. Open Sci. Framew. Available online at: https://osf.io/5nvfe/overview/ (Accessed October 14, 2026).

[B3] Alqaisi O. Al-Ghabeesh S. Masa'la H. Jones A. Joseph K. Tang P. (2026). Nursing roles in early palliative and supportive care for adults with advanced cancer: a scoping review. Curr. Oncol. 33 (6), 312. 10.3390/curroncol33060312 42346212 PMC13298150

[B4] Arksey H. O’Malley L. (2005). Scoping studies: towards a methodological framework. Int. J. Soc. Res. Methodol. 8 (1), 19–32. 10.1080/1364557032000119616

[B5] Barber F. D. (2019). Adverse events of oncologic immunotherapy and their management. Asia-Pacific J. Oncol. Nurs. 6 (3), 212–226. 10.4103/apjon.apjon_6_19 31259216 PMC6518984

[B6] Bray F. Laversanne M. Weiderpass E. Ferlay J. Siegel R. L. Soerjomataram I. (2024). Global cancer statistics 2022: GLOBOCAN estimates of incidence and mortality worldwide for 36 cancers in 185 countries. CA A Cancer J. Clin. 74, 229–263. 10.3322/caac.21834 38572751

[B7] Califano R. Tariq N. Compton S. Fitzgerald D. A. Harwood C. A. Lal R. (2015). Expert consensus on the management of adverse events from EGFR tyrosine kinase inhibitors in the UK. Drugs 75 (12), 1335–1348. 10.1007/s40265-015-0434-6 26187773 PMC4532717

[B8] Chmieliauskaite M. Stojanov I. Saraghi M. Pinto A. (2018). Oral adverse events associated with targeted cancer therapies. Gen. Dent. 66, 26–31. 30188853

[B9] Dailah H. G. Hommdi A. A. Koriri M. D. Algathlan E. M. Mohan S. (2024). Potential role of immunotherapy and targeted therapy in the treatment of cancer: a contemporary nursing practice. Heliyon 10 (2), e24559. 10.1016/j.heliyon.2024.e24559 38298714 PMC10828696

[B10] Diez de los Rios de la Serna C. Boers-Doets C. B. Wiseman T. Radia B. Hammond R. (2024). Early recognition and management of side effects related to systemic anticancer therapy for advanced breast cancer. Semin. Oncol. Nurs. 40 (2), 151553. 10.1016/j.soncn.2023.151553 38171975

[B11] Du R. Wang X. Ma L. Larcher L. M. Tang H. Zhou H. (2021). Adverse reactions of targeted therapy in cancer patients: a retrospective study of hospital medical data in China. BMC Cancer 21 (1), 206. 10.1186/s12885-021-07946-x 33639888 PMC7916285

[B12] Du R. Yang H. Zhou H. Ma L. Getu M. A. Chen C. (2022a). Home-based multidisciplinary interventions on skin adverse reactions in EGFR-TKI-treated patients with lung cancer: a protocol for a randomised controlled trial. BMJ Open 12 (11), e063921. 10.1136/bmjopen-2022-063921 36442902 PMC9710339

[B13] Du R. Yang H. Zhu J. Zhou H. Ma L. Getu M. A. (2022b). Experience of patients with lung cancer and with targeted therapy-related skin adverse drug reactions: a qualitative study. Asia-Pacific Journal Oncology Nursing 9 (10), 100115. 10.1016/j.apjon.2022.100115 36118625 PMC9479359

[B14] Gao Y. Guo M. (2025). Impact of oncology nursing interventions on chemotherapy-induced toxicities in lung cancer patients. Ther. Clin. Risk Manag. 31, 1549–1558. 10.2147/TCRM.S545756 41221162 PMC12599214

[B15] Holcmann M. Sibilia M. (2015). Mechanisms underlying skin disorders induced by EGFR inhibitors. Mol. and Cellular Oncology 2 (4), e1004969. 10.1080/23723556.2015.1004969 27308503 PMC4905346

[B16] Kay P. (2006). Targeted therapies: a nursing perspective. Semin. Oncol. Nurs. InSeminars Oncology Nursing 22, pp. 1–4. 10.1016/j.soncn.2006.01.009 16616280

[B17] Min Y. Zhu Y. Ye M. Zhu Q. Xu Y. Li X. (2023). Life quality improvement of patients with non-small cell lung cancer undergoing targeted therapy: a case study of continuous care. Medicine 102 (44), e35678. 10.1097/MD.0000000000035678 37932985 PMC10627644

[B18] Parreira S. Burns K. Moldawer N. Zomordian N. Bandali N. Virdee K. (2024). The role of nurses in the management of adverse events in patients receiving first-line axitinib plus immuno-oncology agents for advanced renal cell carcinoma. Semin. Oncol. Nurs. InSeminars Oncol. Nurs. 151545. 10.1016/j.soncn.2023.151545 38008655

[B19] Roger S. Edeline J. Campillo-Gimenez B. Ventroux E. Rouge-Bugat M. E. Chapron A. (2020). Adverse events of targeted therapies reported by patients with cancer treated in primary care. Eur. J. Gen. Pract. 26 (1), 202–209. 10.1080/13814788.2020.1846713 33399025 PMC7801082

[B20] Schneider B. J. Naidoo J. Santomasso B. D. Lacchetti C. Adkins S. Anadkat M. (2021). Management of immune-related adverse events in patients treated with immune checkpoint inhibitor therapy: ASCO guideline update. J. Clinical Oncology 39 (36), 4073–4126. 10.1200/JCO.21.01440 34724392

[B21] Shankar A. Wallbridge I. G. Yau C. Saini D. Roy S. Bharati S. J. (2022). Development of management strategies for immune-related adverse effects of immunotherapies used in oncological treatment. Asia-Pacific J. Oncol. Nurs. 9 (1), 7–11. 10.1016/j.apjon.2021.12.007 35528796 PMC9072166

[B22] Sun Y. Y. Wei L. M. Qian Y. (2025). The role of nursing in enhancing quality of life for lung cancer patients receiving targeted and immunotherapy: challenges, opportunities, and future directions. Hum. Vaccines and Immunotherapeutics 21 (1), 2506302. 10.1080/21645515.2025.2506302 40390558 PMC12101592

[B23] Tuominen L. Stolt M. Meretoja R. Leino‐Kilpi H. (2019). Effectiveness of nursing interventions among patients with cancer: an overview of systematic reviews. J. Clinical Nursing 28 (13-14), 2401–2419. 10.1111/jocn.14762 30585667

[B24] Zhang X. Lian T. (2025). Application value of oral targeted drug management program led by specialist nurses in continuous nursing for lung cancer patients. Front. Med. 12, 1693815. 10.3389/fmed.2025.1693815 41346998 PMC12672341

[B25] Zhang J. Yang H. Lu Y. (2024). Management of neurotoxic reactions induced by antibody-drug conjugates. Asia-Pacific J. Oncol. Nurs. 11 (11), 100595. 10.1016/j.apjon.2024.100595 39582554 PMC11582371

