# Supplementary material for: Nursing interventions for managing treatment-related adverse effects of targeted anti-tumor therapies in adult cancer patients: a scoping review
Source: Front Pharmacol. 2026 Oct 20;17:1936674. doi: 10.3389/fphar.2026.1936674 (PMC13727521; doi:10.3389/fphar.2026.1936674)
Supplement: Supplementary file 1 [file Supplementaryfile1.docx]

**Table S1.** Database Search Strings Used in the Scoping Review

| **Database** | **Search String** | **Filters Applied** |
| --- | --- | --- |
| PubMed / MEDLINE | ("nursing"[MeSH Terms] OR "nursing care"[MeSH Terms] OR "oncology nursing"[tiab] OR "nurse-led"[tiab] OR "nursing intervention*"[tiab] OR "nursing management"[tiab]) AND ("targeted therapy"[tiab] OR "targeted anti-tumor therapy"[tiab] OR "tyrosine kinase inhibitor*"[tiab] OR "TKI"[tiab] OR "EGFR inhibitor*"[tiab] OR "monoclonal antibod*"[tiab] OR "immune checkpoint inhibitor*"[tiab] OR "mTOR inhibitor*"[tiab] OR "antibody-drug conjugate*"[tiab] OR "BRAF inhibitor*"[tiab] OR "HER2"[tiab] OR "bevacizumab"[tiab] OR "trastuzumab"[tiab] OR "erlotinib"[tiab] OR "gefitinib"[tiab] OR "osimertinib"[tiab]) AND ("adverse effect*"[tiab] OR "adverse event*"[tiab] OR "toxicity"[tiab] OR "side effect*"[tiab] OR "symptom management"[tiab] OR "toxicity management"[tiab] OR "treatment-related adverse effect*"[tiab]) AND ("adult"[MeSH Terms] OR "adult*"[tiab] OR "cancer patient*"[tiab] OR "oncology patient*"[tiab]) | English language; Publication years: 2015–2025; Humans |
| Scopus | TITLE-ABS-KEY ( ( "nursing" OR "oncology nursing" OR "nurse-led" OR "nursing intervention" OR "nursing management" OR "nursing care" ) AND ( "targeted therapy" OR "targeted anti-tumor therapy" OR "tyrosine kinase inhibitor" OR "EGFR inhibitor" OR "monoclonal antibody" OR "immune checkpoint inhibitor" OR "mTOR inhibitor" OR "antibody-drug conjugate" OR "BRAF inhibitor" OR "TKI" ) AND ( "adverse effect" OR "adverse event" OR "toxicity" OR "side effect" OR "symptom management" OR "toxicity management" ) AND ( "adult" OR "cancer patient" OR "oncology" ) ) AND PUBYEAR > 2014 AND PUBYEAR < 2026 AND LANGUAGE ( english ) | English language; Year: 2015–2025 |
| ScienceDirect | ("nursing intervention" OR "nurse-led" OR "oncology nursing" OR "nursing care" OR "nursing management") AND ("targeted therapy" OR "tyrosine kinase inhibitor" OR "EGFR inhibitor" OR "monoclonal antibody" OR "immune checkpoint inhibitor" OR "mTOR inhibitor" OR "antibody-drug conjugate") AND ("adverse effect" OR "toxicity" OR "adverse event" OR "symptom management") AND ("adult" OR "cancer") | English language; Year: 2015–2025; Journal articles only |
| CINAHL (via EBSCOhost) | (MH "Oncologic Nursing" OR MH "Nursing Care" OR TI "nurse-led" OR AB "nurse-led" OR TI "nursing intervention*" OR AB "nursing intervention*") AND (TI "targeted therapy" OR AB "targeted therapy" OR TI "tyrosine kinase inhibitor*" OR AB "tyrosine kinase inhibitor*" OR TI "EGFR inhibitor*" OR AB "EGFR inhibitor*" OR TI "immune checkpoint inhibitor*" OR AB "immune checkpoint inhibitor*" OR TI "monoclonal antibod*" OR AB "monoclonal antibod*" OR TI "antibody-drug conjugate*" OR AB "antibody-drug conjugate*") AND (TI "adverse effect*" OR AB "adverse effect*" OR TI "toxicity" OR AB "toxicity" OR TI "adverse event*" OR AB "adverse event*" OR TI "symptom management" OR AB "symptom management") | English language; Year: 2015–2025; Peer-reviewed; Human |
| Web of Science | TS=(cancer OR neoplasm*)  AND TS=("targeted therap*" OR "tyrosine kinase inhibitor*" OR "monoclonal antibod*" OR "antibody-drug conjugate*")  AND TS=("nursing intervention*" OR "oncology nurs*" OR "nurse-led")  AND TS=("adverse effect*" OR toxicity OR "adverse event*" OR "symptom management") | Language: English, Article, Review, 2015–2025 |

**Table S2. (PRISMA-ScR) Checklist**

| **SECTION** | **ITEM** | **PRISMA-ScR CHECKLIST ITEM** | **REPORTED ON PAGE #** |
| --- | --- | --- | --- |
| **TITLE** | | | |
| Title | 1 | Identify the report as a scoping review. | Tite page |
| **ABSTRACT** | | | |
| Structured summary | 2 | Provide a structured summary that includes (as applicable): background, objectives, eligibility criteria, sources of evidence, charting methods, results, and conclusions that relate to the review questions and objectives. | Abstract section |
| **INTRODUCTION** | | | |
| Rationale | 3 | Describe the rationale for the review in the context of what is already known. Explain why the review questions/objectives lend themselves to a scoping review approach. | Introduction |
| Objectives | 4 | Provide an explicit statement of the questions and objectives being addressed with reference to their key elements (e.g., population or participants, concepts, and context) or other relevant key elements used to conceptualize the review questions and/or objectives. | Introduction |
| **METHODS** | | | |
| Protocol and registration | 5 | Indicate whether a review protocol exists; state if and where it can be accessed (e.g., a Web address); and if available, provide registration information, including the registration number. | Methods |
| Eligibility criteria | 6 | Specify characteristics of the sources of evidence used as eligibility criteria (e.g., years considered, language, and publication status), and provide a rationale. | Methods |
| Information sources* | 7 | Describe all information sources in the search (e.g., databases with dates of coverage and contact with authors to identify additional sources), as well as the date the most recent search was executed. | Methods |
| Search | 8 | Present the full electronic search strategy for at least 1 database, including any limits used, such that it could be repeated. | Supplementary material |
| Selection of sources of evidence† | 9 | State the process for selecting sources of evidence (i.e., screening and eligibility) included in the scoping review. | Methods |
| Data charting process‡ | 10 | Describe the methods of charting data from the included sources of evidence (e.g., calibrated forms or forms that have been tested by the team before their use, and whether data charting was done independently or in duplicate) and any processes for obtaining and confirming data from investigators. | Methods |
| Data items | 11 | List and define all variables for which data were sought and any assumptions and simplifications made. | Methods |
| Critical appraisal of individual sources of evidence§ | 12 | If done, provide a rationale for conducting a critical appraisal of included sources of evidence; describe the methods used and how this information was used in any data synthesis (if appropriate). | Methods |
| Synthesis of results | 13 | Describe the methods of handling and summarizing the data that were charted. | Methods |
| **RESULTS** | | | |
| Selection of sources of evidence | 14 | Give numbers of sources of evidence screened, assessed for eligibility, and included in the review, with reasons for exclusions at each stage, ideally using a flow diagram. | Results |
| Characteristics of sources of evidence | 15 | For each source of evidence, present characteristics for which data were charted and provide the citations. | Results |
| Critical appraisal within sources of evidence | 16 | If done, present data on critical appraisal of included sources of evidence (see item 12). | NA |
| Results of individual sources of evidence | 17 | For each included source of evidence, present the relevant data that were charted that relate to the review questions and objectives. | Results |
| Synthesis of results | 18 | Summarize and/or present the charting results as they relate to the review questions and objectives. | Results |
| **DISCUSSION** | | | |
| Summary of evidence | 19 | Summarize the main results (including an overview of concepts, themes, and types of evidence available), link to the review questions and objectives, and consider the relevance to key groups. | Discussion |
| Limitations | 20 | Discuss the limitations of the scoping review process. | Limitation |
| Conclusions | 21 | Provide a general interpretation of the results with respect to the review questions and objectives, as well as potential implications and/or next steps. | Conclusion |
| **FUNDING** | | | |
| Funding | 22 | Describe sources of funding for the included sources of evidence, as well as sources of funding for the scoping review. Describe the role of the funders of the scoping review. | Declaration sections |
